# Supplementary material for: FERN – a Java framework for stochastic simulation and evaluation of reaction networks
Source: BMC Bioinformatics. 2008 Aug 29;9:356. doi: 10.1186/1471-2105-9-356 (PMC2553347; doi:10.1186/1471-2105-9-356)
Supplement: Additional file 1 — FERN distribution, Version 1.3. This archive contains the FERN source code and binaries as well as documentation and example models in FernML and SBML. [file 1471-2105-9-356-S1.zip › fern/doc/javadoc/index-files/index-6.html]

F-Index


---


|  |  |  |
| --- | --- | --- |
| **Overview** \| Package \| Class \| Use \| **Tree** \| **Deprecated** \| **Index** \| **Help** | |  |
| **PREV LETTER**   **NEXT LETTER** | **FRAMES**    **NO FRAMES**     **All Classes** |  |


A B C D E F G H I J L M N O P R S T U V 

---


## **F**

**f** - Variable in class fern.simulation.algorithm.AbstractTauLeapingPropensityBoundSimulator: **faculty(int)** - Static method in class fern.tools.NumberTools: Gets the faculty **FeatureNotSupportedException** - Exception in fern.network: **FeatureNotSupportedException(String)** - Constructor for exception fern.network.FeatureNotSupportedException: **fern** - package fern: **fern.analysis** - package fern.analysis: Provides classes and algorithms for analysing networks like ShortestPath, AutocatalyticDetection. **fern.benchmark** - package fern.benchmark: Provides classes for benchmarking simulations (time benchmarks, histogram distance calculation). **fern.cellDesigner** - package fern.cellDesigner: **fern.cellDesigner.ui** - package fern.cellDesigner.ui: **fern.cytoscape** - package fern.cytoscape: Provides the classes for the cytoscape plugin. **fern.cytoscape.ui** - package fern.cytoscape.ui: **fern.example** - package fern.example: Contains examples and demonstrations of the framework. **fern.network** - package fern.network: Provides general classes and interfaces for storing network data. **fern.network.creation** - package fern.network.creation: Provides classes for the evolution of networks. **fern.network.fernml** - package fern.network.fernml: Provides the classes for parsing and using FernML based networks. **fern.network.modification** - package fern.network.modification: Provides classes for modifications of networks. **fern.network.sbml** - package fern.network.sbml: Provides the classes for parsing and using sbml based networks. **fern.simulation** - package fern.simulation: Provides algorithms for simulating reaction network as well as methods for observer certain aspects of a simulation. **fern.simulation.algorithm** - package fern.simulation.algorithm: Provides algorithms for stochastic simultion of reaction networks. **fern.simulation.controller** - package fern.simulation.controller: Provides classes to control the simulations. **fern.simulation.observer** - package fern.simulation.observer: Provides classes to observe certain aspects of simulations. **fern.tools** - package fern.tools: Provides common used tools. **fern.tools.functions** - package fern.tools.functions: Provides common function delegates and implementations. **fern.tools.gnuplot** - package fern.tools.gnuplot: Provides classes to use gnuplot from java. **FernCellDesignerPlugin** - Class in fern.cellDesigner: **FernCellDesignerPlugin()** - Constructor for class fern.cellDesigner.FernCellDesignerPlugin: **FernMLAnnotationManager** - Class in fern.network.fernml: `AnnotationManager` for `FernMLNetwork`s. **FernMLAnnotationManager(Element)** - Constructor for class fern.network.fernml.FernMLAnnotationManager: Create the `AnnotationManager` from the root element of the jdom tree. **FernMLNetwork** - Class in fern.network.fernml: A `FernMLNetwork` is usually loaded from a file. **FernMLNetwork(File)** - Constructor for class fern.network.fernml.FernMLNetwork: Creates a `FernMLNetwork` from a file. **FernMLNetwork(Network)** - Constructor for class fern.network.fernml.FernMLNetwork: Create a `FernMLNetwork` from an existing `Network`. **FernMLNetwork(Network, double[])** - Constructor for class fern.network.fernml.FernMLNetwork: Creates a FernMLNetwork out of an existing network (e.g. to save it to a fernml file) using explicitly given kineticConstants (when net doesn't use `KineticConstantPropensityCalculator` If `kineticConstants` is `null` or to short, a default value of 1 is taken. **FernVisualStyle** - Class in fern.cytoscape: **FernVisualStyle()** - Constructor for class fern.cytoscape.FernVisualStyle: **find(String)** - Static method in class fern.example.ExamplePath: **finished()** - Method in interface fern.analysis.NetworkSearchAction: Gets called, when the search is done. **finished()** - Method in class fern.cytoscape.CytoscapeColorChangeObserver: **finished()** - Method in class fern.example.CellGrowthObserver: **finished()** - Method in class fern.network.sbml.SBMLEventHandlerObserver: **finished()** - Method in class fern.simulation.observer.AmountAtMomentObserver: Do nothing. **finished()** - Method in class fern.simulation.observer.FireTypeObserver: Calculates the average over all runs of the simulator. **finished()** - Method in class fern.simulation.observer.InstantOutputObserver: Prints event. **finished()** - Method in class fern.simulation.observer.IntervalObserver: Adds the recorded data to the average data pool. **finished()** - Method in class fern.simulation.observer.LeapObserver: Do nothing. **finished()** - Method in class fern.simulation.observer.Observer: Gets called when a simulation has finished, directly after the termination check. **finished()** - Method in class fern.simulation.observer.RandomNumberGeneratorCallObserver: Calculates the average. **fireReaction(int, double, Simulator.FireType)** - Method in class fern.simulation.Simulator: Fires a reaction. **fireReaction(int, double, double, int, Simulator.FireType)** - Method in class fern.simulation.Simulator: Fires a reaction. **FireTypeObserver** - Class in fern.simulation.observer: Observes the different types of firings. **FireTypeObserver(Simulator)** - Constructor for class fern.simulation.observer.FireTypeObserver: Create the observer for the given simulator.

---


|  |  |  |
| --- | --- | --- |
| **Overview** \| Package \| Class \| Use \| **Tree** \| **Deprecated** \| **Index** \| **Help** | |  |
| **PREV LETTER**   **NEXT LETTER** | **FRAMES**    **NO FRAMES**     **All Classes** |  |


A B C D E F G H I J L M N O P R S T U V 

---
